# The Development, Deployment, and Evaluation of the CLEFT-Q Computerized Adaptive Test: A Multimethods Approach Contributing to Personalized, Person-Centered Health Assessments in Plastic Surgery

**DOI:** 10.2196/41870

**Published:** 2023-04-27

**Authors:** Conrad Harrison, Inge Apon, Kenny Ardouin, Chris Sidey-Gibbons, Anne Klassen, Stefan Cano, Karen Wong Riff, Andrea Pusic, Sarah Versnel, Maarten Koudstaal, Alexander C Allori, Carolyn Rogers-Vizena, Marc C Swan, Dominic Furniss, Jeremy Rodrigues

**Affiliations:** 1 Nuffield Department of Orthopaedics, Rheumatology and Musculoskeletal Sciences University of Oxford Oxford United Kingdom; 2 Department of Oral and Maxillofacial Surgery Dutch Craniofacial Center Erasmus University Medical Center Rotterdam Netherlands; 3 Cleft Lip and Palate Association London United Kingdom; 4 Department of Psychology, Speech and Hearing University of Canterbury Christchurch New Zealand; 5 MD Anderson Center for INSPiRED Cancer Care University of Texas Houston, TX United States; 6 Department of Pediatrics McMaster University Hamilton, ON Canada; 7 Modus Outcomes Letchworth Garden City United Kingdom; 8 Department of Plastic and Reconstructive Surgery Hospital for Sick Children Toronto, ON Canada; 9 Patient-Reported Outcomes, Values & Experience Center Department of Surgery, Brigham and Women’s Hospital Harvard Medical School Boston, MA United States; 10 Department of Plastic and Reconstructive Surgery Dutch Craniofacial Center Erasmus University Medical Center Rotterdam Netherlands; 11 Division of Plastic, Maxillofacial & Oral Surgery Duke University Hospital & Children's Health Center Durham, NC United States; 12 Department of Plastic and Oral Surgery Boston Children’s Hospital Boston, MA United States; 13 The Spires Cleft Centre, John Radcliffe Hospital Oxford University Hospitals Oxford United Kingdom; 14 Warwick Clinical Trials Unit Warwick Medical School University of Warwick Coventry United Kingdom; 15 Department of Plastic Surgery Stoke Mandeville Hospital Buckinghamshire Healthcare NHS Trust Aylesbury United Kingdom

**Keywords:** cleft lip, cleft palate, patient-reported outcome measures, outcome assessment, CLEFT-Q, computerized adaptive test, CAT

## Abstract

**Background:**

Routine use of patient-reported outcome measures (PROMs) and computerized adaptive tests (CATs) may improve care in a range of surgical conditions. However, most available CATs are neither condition-specific nor coproduced with patients and lack clinically relevant score interpretation. Recently, a PROM called the CLEFT-Q has been developed for use in the treatment of cleft lip or palate (CL/P), but the assessment burden may be limiting its uptake into clinical practice.

**Objective:**

We aimed to develop a CAT for the CLEFT-Q, which could facilitate the uptake of the CLEFT-Q PROM internationally. We aimed to conduct this work with a novel patient-centered approach and make source code available as an open-source framework for CAT development in other surgical conditions.

**Methods:**

CATs were developed with the Rasch measurement theory, using full-length CLEFT-Q responses collected during the CLEFT-Q field test (this included 2434 patients across 12 countries). These algorithms were validated in Monte Carlo simulations involving full-length CLEFT-Q responses collected from 536 patients. In these simulations, the CAT algorithms approximated full-length CLEFT-Q scores iteratively, using progressively fewer items from the full-length PROM. Agreement between full-length CLEFT-Q score and CAT score at different assessment lengths was measured using the Pearson correlation coefficient, root-mean-square error (RMSE), and 95% limits of agreement. CAT settings, including the number of items to be included in the final assessments, were determined in a multistakeholder workshop that included patients and health care professionals. A user interface was developed for the platform, and it was prospectively piloted in the United Kingdom and the Netherlands. Interviews were conducted with 6 patients and 4 clinicians to explore end-user experience.

**Results:**

The length of all 8 CLEFT-Q scales in the International Consortium for Health Outcomes Measurement (ICHOM) Standard Set combined was reduced from 76 to 59 items, and at this length, CAT assessments reproduced full-length CLEFT-Q scores accurately (with correlations between full-length CLEFT-Q score and CAT score exceeding 0.97, and the RMSE ranging from 2 to 5 out of 100). Workshop stakeholders considered this the optimal balance between accuracy and assessment burden. The platform was perceived to improve clinical communication and facilitate shared decision-making.

**Conclusions:**

Our platform is likely to facilitate routine CLEFT-Q uptake, and this may have a positive impact on clinical care. Our free source code enables other researchers to rapidly and economically reproduce this work for other PROMs.

## Introduction

Patient-reported outcome measures (PROMs) have gained widespread acceptance as tools for measuring the impact of treatments on elements of health that matter most to patients. There is also a rapidly growing body of evidence to suggest that adopting PROM feedback into surgical care improves outcomes by enhancing clinical communication and facilitating detection of previously unidentified issues. For many conditions, the use of PROMs is associated with improved health-related quality of life (HRQOL), faster detection of clinical deterioration, and even improved survival [1-4]. PROMs may be especially helpful in pediatric surgical care, where they may also deliver improved communication, more sensitive detection of HRQOL issues, higher referral rates, better patient experience, and faster consultations [5-10].

A key group that would benefit from the routine use of PROMs are those with cleft lip or palate (CL/P) and other craniofacial conditions. CL/P is one of the most common birth differences, affecting 1 in 700 internationally, with significant implications for a person’s facial appearance, dentition, speech, psychosocial development, and education [11]. The International Consortium for Health Outcomes Measurement (ICHOM) has recently proposed a Standard Set of outcome measures for the “comprehensive appraisal of cleft care,” which largely comprises scales (questionnaires) from the CLEFT-Q, a condition-specific PROM for people aged 8 to 29 years, born with a CL/P or other craniofacial conditions [12,13].

The 8 CLEFT-Q scales are included in the ICHOM Standard Set for CL/P measure: the appearance of the face, teeth, and jaws; speech function and speech distress; and school, social, and psychological function. These scales contain between 7 and 12 items (questions), equating to 76 items when all 8 scales are administered simultaneously [12,14].

Barriers to using PROMs such as CLEFT-Q in routine surgical practice include delays in obtaining scores, scores that are difficult to interpret, reference ranges that are difficult to interpret, and difficulties in data governance [15]. In addition, response burden may be an important barrier to implementing PROMs in pediatric settings as it is not always appropriate to administer lengthy assessments to young children in clinical practice. This has limited the uptake of the CLEFT-Q and ICHOM Standard Set for CL/P internationally [16-19].

Computerized adaptive tests (CATs) are a potential way to overcome these barriers. CATs use algorithms that can make PROMs like CLEFT-Q shorter and more personalized by selecting the most relevant questions for an individual, based on the answers that a person has already provided during the assessment. There are 3 components to a basic CAT algorithm: a score estimator, an item selection criterion, and a stopping rule. The score estimator predicts a person’s score from the responses obtained so far during the assessment. The item selection criterion then selects the most useful question to ask, based on the score estimate. This approach avoids asking questions that are unlikely to improve measurement precision. To illustrate, consider an assessment of mobility. If we know that a patient has difficulty walking 100 m, it would not be helpful to ask whether they have difficulty walking a mile. Instead, a CAT algorithm may select a question more appropriately targeted to that patient, for example whether they have difficulty walking from room to room in their house unaided. The stopping rule terminates the assessment when a prespecified criterion is met, for example after a certain number of questions or a given level of measurement precision. This individually tailored approach balances a PROM’s reliability with its length to reduce response burden and is hoped to improve PROM uptake, in both routine clinical practice and research.

There are notable limitations to available CAT platforms in clinical surgery. First, most surgical CATs are generic (as opposed to condition-specific) measures, which may fail to adequately capture the elements of health most important to patients with specific health needs [20]. Second, CAT scores are often interpreted through comparison with general population scores. A more useful approach may be to compare a person’s score with the scores of people who have similar demographic and clinical characteristics [21]. Third, the number of questions in most CATs is chosen based on psychometric heuristics relating to the assessment’s standard error of measurement, which is an indicator of theoretical measurement reliability [22]. Finally, most CATs send a person’s response from the electronic health records (EHRs) platform to an external assessment center to select the next question. This is less efficient and secure than a locally implemented system [23].

The aim of this project was to address these barriers and limitations with a novel system that can deploy person-centered CATs for the CLEFT-Q scales, and feed scores back to clinicians and patients in a rapid, engaging, and clinically useful way. We designed the platform to be open-source and transferrable so that it could be easily, cheaply, and rapidly adapted for any surgical PROM meeting contemporary psychometric standards.

## Methods

### CAT Calibration

We developed CAT algorithms for each CLEFT-Q scale in the ICHOM Standard Set using responses to full-length CLEFT-Q scales that were obtained from the CLEFT-Q field test. This study recruited from October 2014 to November 2016 and collected CLEFT-Q responses from 2434 participants aged 8 to 29 years from 30 cleft treatment units in 12 countries. The participants in the CLEFT-Q field test were at a variety of treatment stages for either isolated cleft lip (CL), isolated cleft palate (CP), cleft lip and alveolus (CLA), or cleft lip, alveolus, and palate (CLAP). Patients with a CL were not asked to complete Speech Function or Speech Distress scales, only children currently in schools were asked to complete the School Function scale, and only participants aged 12 years and older were asked to complete the Jaw scale. Each respondent in this cohort completed the CLEFT-Q at 1 time point. Local Institutional Review Board approval was obtained from each center. An in-depth report describing the methodology and results of the CLEFT-Q field test has been published previously [14].

We performed Rasch analysis in R to calibrate CAT parameters from these data (see Rasch Parameterization, Multimedia Appendix 1 [24-28]). Rasch analysis is a framework for the development and evaluation of statistical models that describe the relationship between a person’s level of measured construct and the probability that they will endorse a certain item response. For example, in the CLEFT-Q social function scale, Rasch models explain how likely a person is to respond to an item in a given way, based on their overall social function level. These models are used by CAT algorithms to estimate a person’s overall score, and also to select the most useful item to pose, given the current score estimate. Specific CAT settings for score calculation and item selection were chosen based on the previous optimization studies [29].

### CAT Validation

We evaluated the performance of these CAT algorithms in an independent validation data set that included the CLEFT-Q responses of 536 participants, during 561 clinic appointments. These were collected between November 2015 and April 2019 at Erasmus University Medical Center, the Netherlands, as well as Boston Children’s Hospital and Duke Children’s Hospital, both in the United States. Respondents were aged 7 to 24 years and receiving care for either CL, CP, CLA, or CLAP. The timing of scale administration approximately followed the recommendations proposed in the ICHOM Standard Set: Clinical teams aimed to assess patients at 8 years of age with the CLEFT-Q face, teeth, and social function scales, and then again at approximately 12 and 22 years of age with scales that were pertinent to the patient’s specific cleft type. For example, a 22-year-old with an isolated CP would complete the face, jaws, teeth, speech distress, speech function, and social function scales. Incomplete response sets were removed via listwise exclusion and outliers were determined by Mahalanobis distance (see *Missing Data and Outliers in the Validation Dataset* in Multimedia Appendix 1).

We ran a series of Monte Carlo simulations in which CAT algorithms aimed to estimate the full-length CLEFT-Q scale scores of each participant in the validation data set, based on a predetermined number of their item responses, using an R package that we developed specifically for this study [30]. For example, the CAT for the CLEFT-Q face scale (9 items) first aimed to estimate each respondent’s CLEFT-Q face score from all 9 items, then from 8 items only, and then again from 7 items. The algorithms used Bayesian statistics to choose which items to administer, and in which order (see Computerized Adaptive Test Simulation Settings, Multimedia Appendix 1). For each CAT, at each possible assessment length, concordance between CAT and full-length score was measured with the Pearson correlation coefficient, root-mean-square error (RMSE), and 95% limits of agreement. RMSE is a measure of the difference between full-length CLEFT-Q scale scores and CLEFT-Q CAT scores, averaged across the population, and 95% limits of agreement demonstrate the difference between full-length CLEFT-Q scale scores and CLEFT-Q CAT scores at the individual level. For example, if the 95% limits of agreement between full-length and CAT scores were 7.00 to +7.00, we would expect that 95% of the time, for any individual, the CAT score would fall within 7.00 points of the full-length scale score.

In secondary sensitivity analyses, these computations were repeated with and then without outliers, and with both listwise inclusion and imputation of missing item responses (see *Missing Data and Outliers in the Validation Dataset* in Multimedia Appendix 1).

### Multistakeholder Consensus Workshop

We discussed the findings of the validation study during a multistakeholder consensus workshop attended by 3 adults who were born with a CL/P, 5 current patients aged 11-16 years (accompanied by 1 parent each), 2 psychologists, 2 cleft surgeons, 2 speech and language therapists, 1 dentist, 1 orthodontist, and 2 cleft specialist nurses. Prior to the workshop, participants were asked to read through the full-length CLEFT-Q.

For each scale, the balance between accuracy and burden was discussed in web-based breakout rooms with experienced facilitators ensuring that all voices were heard. Particular consideration was given to the scale length, the item wordings, participants’ experiences of administering or completing the questionnaire, and the results of the validation study. Every participant voted on the assessment length they felt was most appropriate for each scale. CAT assessment lengths were chosen based on majority voting at this workshop.

### User Interface Development

We built a user interface to administer each CLEFT-Q scale according to its respective CAT algorithm, using the Concerto platform [31]. Concerto can run CAT algorithms internally, and be installed locally, such that CATs can be administered via Concerto without data leaving a hospital’s local server. We integrated the results into a Shiny app that we have called the Score Checker, to help patients and clinicians visualize and interpret CLEFT-Q CAT scores within the clinical context.

### Pilot Testing

The CLEFT-Q CAT platform was tested in outpatient cleft clinics in Oxford (United Kingdom) and Rotterdam (the Netherlands). Patients were asked to complete relevant CLEFT-Q CAT scales in the waiting room, prior to their clinical appointment. Scores were then reviewed by the clinical team before the patient entered the consultation room. Clinicians were then free to discuss and action these results as appropriate in the clinical situation.

A purposively diverse sample of UK patients and clinicians that had used the platform within the last 7 days were recruited for semistructured interviews that explored the platform’s user experience. It was important to interview both patients and clinicians, as the platform is intended to be acceptable, usable, and of benefit to both of these stakeholder groups. The selection of patients for interviewing was made to be deliberately diverse by age, gender, cleft type, and ethnicity. The selection of clinicians was deliberately diversified by gender and occupation.

Interviews were recorded and transcribed verbatim and then coded with the NVivo platform (version 1.0 for Mac) under the following prespecified categories: experience of the CAT’s content; experience of the software; barriers to implementing the CLEFT-Q CAT; and facilitators to implementing the CLEFT-Q CAT. Emergent themes within and outside these categories were synthesized through an inductive approach. Clinicians involved in piloting the platform at both sites reviewed these themes to check that they accurately and comprehensively captured their experience. A completed Consolidated Criteria for Reporting Qualitative Research (ie, COREQ) checklist [32] is provided in Supplementary Table 1 (Multimedia Appendix 1). This provides a detailed and standardized report of the qualitative element to this work, including information about the research team, study design, and analysis. Interview schedules are provided in Supplementary Tables 2 and 3 (Multimedia Appendix 1).

### Ethics Approval

Ethical approval for this work was obtained from the University of Oxford Medical Sciences Interdivisional Research Ethics Committee (R74005/RE001).

## Results

### Demographics

The clinical and demographic variables for the CAT calibration and validation datasets are presented in Table 1. Within both data sets, there was a preponderance toward the male sex and a diagnosis of CLAP.

Table 2 summarizes the correlation and agreement between CAT scores and full-length assessments for each scale in the validation data set. As the number of items in a CAT decreased, so did the correlation and agreement of CAT and full-length scale scores (Table 2). A decrease in scale length of 2 items did not significantly affect accuracy, with correlation coefficients of 0.97 or above for all and RMSE ranging from 2 to 5 at this level of item reduction.

**Table 1:** Clinical and demographic variables of the calibration and validation data sets for each computerized adaptive test.

				Psychological		Social		School		Speech distress		Speech function		Face		Teeth		Jaws
**Calibration data set**																		
	Included participants, n			2187		2154		1527		1819		1764		2301		2227		1443
	**Age**																	
		Age (years), median (IQR)	14 (7)		14 (7)		12 (5)		14 (7)		14 (7)		14 (7)		14 (7)		16 (5)	
		Missing data, n	1		0		0		0		0		1		0		0	
	**Gender, n**																	
		Male	1217		1199		866		1007		973		1277		1231		775	
		Female	968		954		661		812		791		1022		995		667	
		Missing data	0		0		0		0		0		0		0		0	
	**Patients by country, n**																	
		Australia	23		24		15		20		20		23		25		12	
		Canada	476		468		260		380		369		592		526		345	
		England	312		304		233		263		253		309		309		205	
		Ireland	95		93		57		87		90		96		96		79	
		United States	354		351		312		317		316		350		348		178	
		The Netherlands	197		195		129		160		153		198		194		138	
		India	231		232		176		174		172		232		231		106	
		Sweden	93		91		80		77		71		93		92		62	
		Turkey	54		52		36		47		50		54		54		49	
		Colombia	180		174		105		148		119		183		184		137	
		Chile	84		81		57		74		76		84		85		71	
		Spain	88		89		67		72		75		87		83		61	
		Missing	0		0		0		0		0		0		0		0	
	**Cleft type, n**																	
		Cleft lip	244		233		175		0		0		252		248		146	
		Cleft palate	494		493		374		482		464		526		514		301	
		Cleft lip and alveolus	179		178		139		128		127		195		191		122	
		Cleft lip, alveolus, and palate	1270		1250		839		1209		1173		1328		1274		874	
		Missing data	0		0		0		0		0		0		0		0	
**Validation data set**																		
	Included participants, n			247		345		247		258		274		530		529		314
	**Age**																	
		Age (years), median (IQR)	12 (1)		9 (5)		12 (1)		12 (5)		12 (5)		11 (3)		11 (3)		12 (10)	
		Missing data, n	0		0		0		0		0		0		0		0	
	**Gender, n**																	
		Male	134		189		134		134		144		292		290		164	
		Female	113		156		113		124		130		238		239		150	
		Missing data	0		0		0		0		0		0		0		0	
	**Patients by country, n**																	
		The Netherlands	130		226		130		157		174		354		358		214	
		United States	117		119		117		101		100		176		171		100	
		Missing	0		0		0		0		0		0		0		0	
	**Cleft type, n**																	
		Cleft lip	13		27		13		4		4		39		40		22	
		Cleft palate	71		99		70		86		93		151		151		94	
		Cleft lip and alveolus	24		29		24		7		7		51		50		29	
		Cleft lip, alveolus, and palate	139		190		140		161		170		289		288		169	
		Missing data	0		0		0		0		0		0		0		0	

**Table 2:** Computerized adaptive test (CAT) performance in the validation data set.^a^

Scale and CAT length (items)		Correlation with full length	Root-mean-square error	Lower 95% limit of agreement	Upper 95% limit of agreement
**Face (9 items in total)**					
	8	0.997	1.67	–3.52	2.92
	7^b^	0.989^b^	3.19^b^	–6.80^b^	5.46^b^
	6	0.983	4.01	-8.48	7.00
	5	0.972	5.07	–10.41	9.35
**Jaw (7 items in total)**					
	6^b^	0.997^b^	2.24^b^	–4.09^b^	4.64^b^
	5	0.992	3.68	–6.82	7.57
	4	0.985	5.23	–9.70	10.72
	3	0.980	6.28	–11.68	12.86
**Teeth (8 items in total)**					
	7	0.995	2.14	–3.87	4.46
	6^b^	0.989^b^	3.17^b^	–6.33^b^	6.12^b^
	5	0.982	4.13	–8.20	7.98
	4	0.968	5.47	–10.74	10.74
**School (10 items in total)**					
	9	0.996	2.00	–4.20	3.54
	8	0.991	2.92	–6.07	5.28
	7^b^	0.987^b^	3.53^b^	–7.28^b^	6.52^b^
	6	0.975	4.97	–10.26	9.12
**Psychological function (10 items in total)**					
	9	0.997	1.98	–3.70	4.03
	8^b^	0.994^b^	2.72^b^	–5.27^b^	5.41^b^
	7	0.989	3.75	–7.20	7.53
	6	0.985	4.45	–8.23	9.15
**Speech distress (10 items in total)**					
	9^b^	0.995^b^	2.15^b^	–4.48^b^	3.85^b^
	8	0.973	5.44	–11.82	8.58
	7	0.947	7.61	–16.58	11.79
	6	0.904	10.44	–22.82	15.75
**Speech function (12 items total)**					
	11	0.998	1.79	–3.86	2.90
	10	0.992	3.10	–6.59	5.31
	9	0.987	4.14	–8.91	6.83
	8^b^	0.981^b^	4.98^b^	–10.75^b^	8.12^b^
**Social function (10 items in total)**					
	9	0.998	1.40	–2.88	2.57
	8^b^	0.995^b^	2.16^b^	–4.14^b^	4.32^b^
	7	0.988	3.45	–7.19	6.22
	6	0.984	4.08	–8.61	7.19

^a^Correlation between linear assessment and CAT scores; the root-mean-square error was calculated between the linear assessment and CAT scores (out of 100 points); and the 95% limit of agreement was determined between the linear assessment and CAT scores (out of 100 points) in accordance with the Bland-Altman method.

^b^CAT settings selected by stakeholders to represent the optimal balance between accuracy and assessment burden.

Exclusion of outliers and imputation of missing data did not significantly affect these results. Complete results tables, including those of the sensitivity analyses, are available in sheets 4 and 5 of Multimedia Appendix 2.

### Multistakeholder Workshop

The CAT lengths that were chosen to represent the optimal balance between accuracy and burden during the multistakeholder workshop are indicated in Table 2 and sheet 6 of Multimedia Appendix 2. The RMSE of these CATs ranged from 2 to 5 points out of 100 from the full-length assessment scores.

### User Interface

**Figure 1:**
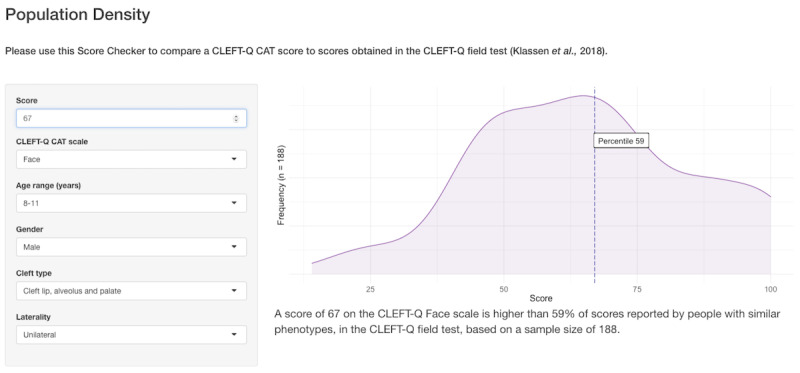
Population density tab of the Score Checker web application.

Figure 1 demonstrates the population density tab of the Score Checker app. Scores are expressed as a percentile of CLEFT-Q field test scores from respondents with similar demographics (age, gender, cleft type, and laterality). In the left panel, the users can filter the CLEFT-Q field test data based on clinical and demographic variables. The magenta density plot demonstrates the distribution of scores achieved by individuals after filtering, with sample sizes displayed on the y-axis and in text below the plot. The vertical, blue dashed line superimposing the plot demonstrates where a given score would fall in this distribution.

**Figure 2:**
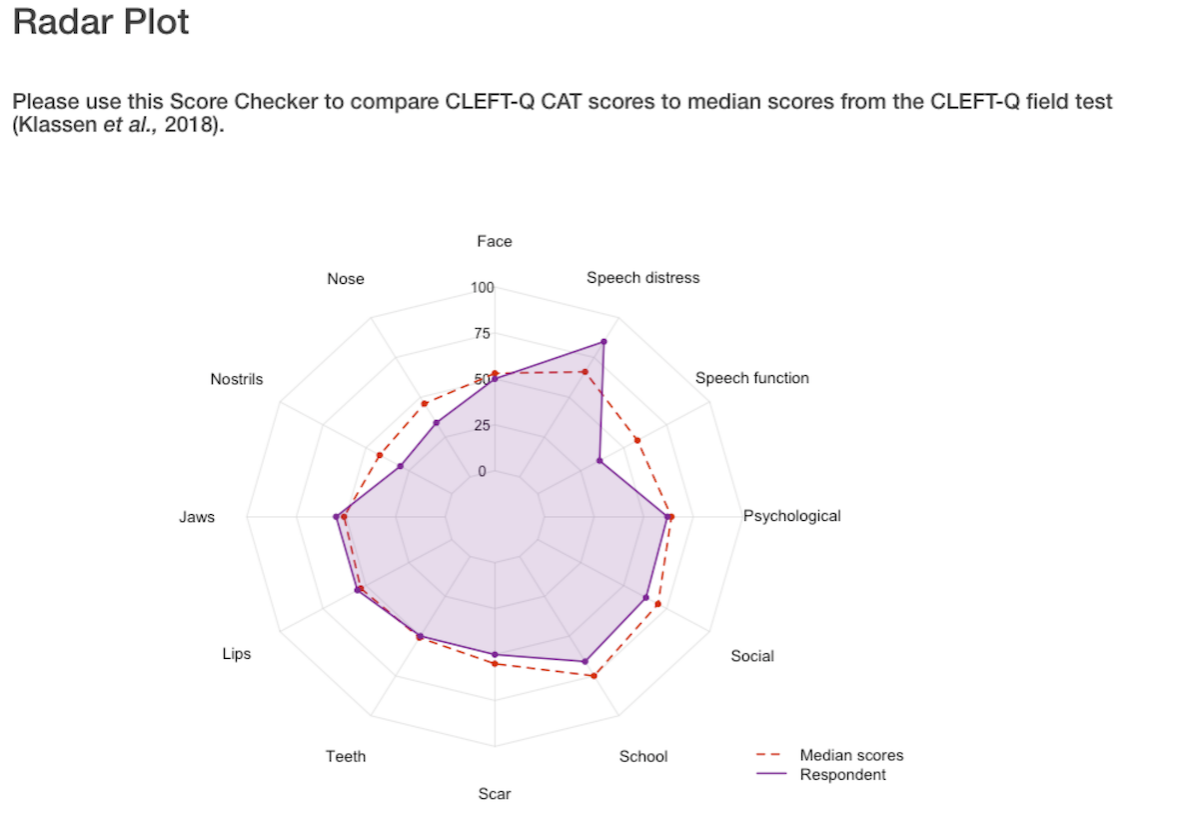
Radar plot tab of the Score Checker web application.

Figure 2 demonstrates the output of the Radar plot tab of the Score Checker app. The magenta points represent an individual patient’s scores, and the red points are median field test scores from respondents with similar demographics, based on the filters applied (see the left panel of Figure 1). Outermore points indicate higher (clinically better) CLEFT-Q scores. The illustrations of the patient-facing interface are provided in the Supplementary Figure 1 and Supplementary Figure 2, Multimedia Appendix 1.

### Semistructured Interviews

We recruited 6 patients and 4 clinicians for semistructured interviews. This included 3 male and 3 female patients aged 8 to 28 years with a variety of diagnoses and ethnicities (see Supplementary Table 4 in Multimedia Appendix 1 for participant characteristics), and a cleft surgeon, a cleft specialist nurse, a speech therapist, and a dentist.

Positive themes relating to the content of the CAT included its ability to cause patients to think about previously unconsidered health aspects, its person-centeredness, and its ability to normalize health concerns. Negative themes relating to the CAT content were repetitiveness and its potential to cause upset to patients who would rather not answer sensitive questions. Themes relating to the user interface were its ease of use and a preference for electronic tablets over pen and paper. No participant felt the CAT caused excessive response burden, even when asked directly. The quotes to illustrate these themes are provided in *Thematic Analysis* in Multimedia Appendix 1.

Potential barriers to implementing the system included integration across different EHR platforms, maintaining equality of care between hub and spoke services, a physical means of collecting data (eg, electronic tablets, staffing, and space), opportunity costs for patients and clinicians, reluctance to use technology, and change resistance. Facilitators included the opportunistic use of waiting room time, training, and education of the benefits. The option to complete the assessment at home was seen as a facilitator by some but not by others.

The use of the CAT as a clinical communication aid was an emergent theme within both patient and clinician interviews. Subthemes, illustrated in Table 3, included improving consultation focus; improving patient-to-clinician information flow; facilitating a multidisciplinary approach to care; improving patient readiness; and facilitating shared decision-making.

**Table 3:** Subthemes relating to the use of the CLEFT-Q computerized adaptive test as a communication aid.

Subtheme	Participants reporting subtheme	Example quotes
Improving consultation focus	2 clinicians	“We had a patient where they have cleft as part of a complex craniofacial condition, and there had been planning and suggestion of doing really quite major surgery, reconstructing around the nose area, but when the patient did the CAT before they came into the clinic, actually that was one of their lowest priorities, which meant that we could then refocus the consultation to focus on their priorities rather than what had been perceived as what should be discussed.” [clinician]
Improving patient-to-clinician information flow	2 patients and 2 clinicians	“I was forearmed and so forewarned so I could broach things differently with her [my patient], have a slightly different dialogue and then facilitate some supportive therapy with clinical psychologists.” [clinician] “I found the questionnaire really helped me be more honest about what was bothering me.” [patient]
Facilitating a multi-disciplinary approach to care	2 clinicians	“I think what it allows patients to do, is be seen as a whole patient rather than just an element of treatment.” [clinician] “All of the patient’s focus was on his teeth and jaws and I perhaps wouldn't have been thinking very much about that in my own sort of uni-disciplinary way, and it meant really that, you know, it's [the CLEFT-Q CAT is] actually guiding the treatment pathway for him.” [clinician]
Improving patient readiness	2 patients and 3 clinicians	“It made me more alert as to why I was there.” [patient]
Facilitating shared decision-making	1 patient and 2 clinicians	“What it [the CLEFT-Q CAT] does do is open doors for patients and clinicians to rethink the direction sometimes they were taking [in their care plan].” [clinician] “It helps set the plan of what’s more important and what we can do first [which heath interventions should be prioritized].” [patient]

## Discussion

### Principal Findings

We have developed, validated, deployed, and evaluated a system that can facilitate the uptake of high-quality, standardized outcome measurement for CL/P and other craniofacial conditions, and act as an open-source framework for the development of other surgical CATs. Our approach to CAT development has focused on person-centeredness, and elements of our methodology may be preferable to those used in popular alternatives. First, we have coproduced our software with people who are undergoing, or have undergone, treatment for the condition of interest. We included patients in the setting of CAT stopping rules, rather than deciding the acceptable level of response burden on their behalf. Second, the platform uses condition-specific measures, administered at fixed lengths chosen by stakeholders, and presents scores in comparison to clinically relevant populations (see Figure 1). In practice, we anticipate this translating into patients being more likely to complete our CAT than others developed with conventional methods that do not include patients. The platform can also run locally without internet access, meaning that data never have to be shared outside the clinical environment. This may make our system more efficient and more secure than alternative platforms.

It is possible that these design elements will directly facilitate PROM uptake, as individualization of PROMs, assessment burden, and interpretability of results have all been identified as important “pinch points” for the PROM implementation pipeline [33]. We have made source code for our validation software and Score Checker app freely available for open appraisal and reproduction [30,34]. These can be quickly and cost-effectively translated into other outcome measurement systems.

Our thematic analysis suggests that the platform encourages patient reflection, improves the patient-to-clinician information flow, and facilitates clinical prioritization and shared decision-making. These findings are consistent with frameworks derived from other qualitative research into PROM implementation [10,33,35]. Although patients found the content of the CAT person-centered, it was also described as repetitive. This may be a generalizable finding of CATs for PROMs, as they aim to select the best-targeted (most salient) items from a scale, which may be similar in content to each other. Although response burden is a well-described barrier to CLEFT-Q implementation [16-19], none of our interview participants felt that the CAT was excessively burdensome, even on direct questioning: “for me it was pretty quick, so anyone could fill in this form” [patient].

There are some limitations to this work. The CLEFT-Q is a novel instrument, and there are no longitudinal, anchor-based estimates for CLEFT-Q scales’ minimal important change or minimal important difference. This means that our system is limited to interpreting a patient’s score through comparison with cross-sectional data from matched populations, using, for example, median scores. When a change has occurred in an individual (eg, following treatment), it is difficult to relate this to real-world change that is meaningful to the patient. Similarly, it is difficult to confidently say whether 1 treatment or 1 hospital achieves meaningfully better results than another. Ongoing work into CLEFT-Q interpretability, driven partly by ICHOM’s promotion of the PROM, will support our platform’s use in long-term monitoring and interdepartmental benchmarking.

Future work will look to address these limitations and the other implementation barriers described in our thematic synthesis. The extent to which clinical PROM integration improves patient outcomes in CL/P and other complex, long-term surgical conditions should also be explored in future research. The existing frameworks suggest that they may be most impactful as screening tools, clinical monitoring tools, and decision support systems for shared care planning [33], and this is consistent with our findings.

### Conclusions

We have provided an open-source framework for the development of condition-specific, person-centered CAT platforms, and used this to develop and implement a CAT for the CLEFT-Q. This novel approach may be more person-centered and clinically useful than alternatives. The platform was perceived to improve clinical communication and patient experience, and will facilitate the implementation of routine, standardized PROMs in CL/P care. Our methods are generalizable to other long-term, multisystem conditions. We have provided all necessary material for researchers to reproduce these tools for other PROMs.

## Appendix

Multimedia Appendix 1 Supplementary Appendix detailing methods and results.

Multimedia Appendix 2 Supplementary results tables, including missing item responses for each scale in the calibration dataset (Sheet 1), Rasch model parameters and fit statistics (Sheet 2), outliers in the validation dataset (Sheet 3), computerized adaptive testing results in logits (Sheet 4) and as transformed 0-100 scores (Sheet 5), and multistakeholder workshop vote results (Sheet 6).

## Abbreviations

CAT: computerized adaptive test
CL: cleft lip
CLA: cleft lip and alveolus
CLAP: cleft lip, alveolus, and palate
CL/P: cleft lip or palate
CP: cleft palate
EHR: electronic health record
HRQOL: health-related quality of life
ICHOM: International Consortium for Health Outcomes Measurement
PROM: patient-reported outcome measure
RMSE: root-mean-square error

## References

[1] Denis F, Lethrosne C, Pourel N, Molinier O, Pointreau Y, Domont J, Bourgeois H, Senellart H, Trémolières P, Lizée T, Bennouna J, Urban T, El Khouri C, Charron A, Septans A, Balavoine M, Landry S, Solal-Céligny P, Letellier C (2017). Randomized trial comparing a web-mediated follow-up with routine surveillance in lung cancer patients. J Natl Cancer Inst.

[2] Basch E, Deal AM, Kris MG, Scher HI, Hudis CA, Sabbatini P, Rogak L, Bennett AV, Dueck AC, Atkinson TM, Chou JF, Dulko D, Sit L, Barz A, Novotny P, Fruscione M, Sloan JA, Schrag D (2016). Symptom monitoring with patient-reported outcomes during routine cancer treatment: a randomized controlled trial. J Clin Oncol.

[3] Strasser F, Blum D, von Moos R, Cathomas R, Ribi K, Aebi S, Betticher D, Hayoz S, Klingbiel D, Brauchli P, Haefner M, Mauri S, Kaasa S, Koeberle D, Swiss Group for Clinical Cancer Research (SAKK) (2016). The effect of real-time electronic monitoring of patient-reported symptoms and clinical syndromes in outpatient workflow of medical oncologists: E-MOSAIC, a multicenter cluster-randomized phase III study (SAKK 95/06). Ann Oncol.

[4] Stuck AE, Moser A, Morf U, Wirz U, Wyser J, Gillmann G, Born S, Zwahlen M, Iliffe S, Harari D, Swift C, Beck JC, Egger M (2015). Effect of health risk assessment and counselling on health behaviour and survival in older people: a pragmatic randomised trial. PLoS Med.

[5] Engelen V, Detmar S, Koopman H, Maurice-Stam H, Caron H, Hoogerbrugge P, Egeler RM, Kaspers G, Grootenhuis M (2012). Reporting health-related quality of life scores to physicians during routine follow-up visits of pediatric oncology patients: is it effective?. Pediatr Blood Cancer.

[6] Engelen V, van Zwieten M, Koopman H, Detmar S, Caron H, Brons P, Egeler M, Kaspers G, Grootenhuis M (2012). The influence of patient reported outcomes on the discussion of psychosocial issues in children with cancer. Pediatr Blood Cancer.

[7] Wolfe J, Orellana L, Cook EF, Ullrich C, Kang T, Geyer JR, Feudtner C, Weeks JC, Dussel V (2014). Improving the care of children with advanced cancer by using an electronic patient-reported feedback intervention: results from the PediQUEST randomized controlled trial. J Clin Oncol.

[8] Murillo M, Bel J, Pérez J, Corripio R, Carreras G, Herrero X, Mengibar J, Rodriguez-Arjona D, Ravens-Sieberer U, Raat H, Rajmil L (2017). Impact of monitoring health-related quality of life in clinical practice in children with type 1 diabetes mellitus. Qual Life Res.

[9] Haverman L, Engelen V, van Rossum MA, Heymans HS, Grootenhuis MA (2011). Monitoring health-related quality of life in paediatric practice: development of an innovative web-based application. BMC Pediatr.

[10] Bele S, Chugh A, Mohamed B, Teela L, Haverman L, Santana MJ (2020). Patient-reported outcome measures in routine pediatric clinical care: a systematic review. Front Pediatr.

[11] Mossey PA, Little J, Munger RG, Dixon MJ, Shaw WC (2009). Cleft lip and palate. Lancet.

[12] Allori AC, Kelley T, Meara JG, Albert A, Bonanthaya K, Chapman K, Cunningham M, Daskalogiannakis J, de Gier H, Heggie AA, Hernandez C, Jackson O, Jones Y, Kangesu L, Koudstaal MJ, Kuchhal R, Lohmander A, Long RE, Magee L, Monson L, Rose E, Sitzman TJ, Taylor JA, Thorburn G, van Eeden S, Williams C, Wirthlin JO, Wong KW (2017). A standard set of outcome measures for the comprehensive appraisal of cleft care. Cleft Palate Craniofac J.

[13] Klassen AF, Rae C, Wong Riff KW, Bulstrode N, Denadai R, Goldstein J, Hol ML, Murray DJ, Bracken S, Courtemanche DJ, O'Hara J, Butler D, Tassi A, Malic CC, Ganske IM, Phua YS, Marucci DD, Johnson D, Swan MC, Breuning EE, Goodacre TE, Pusic AL, Cano S (2021). FACE-Q craniofacial module: part 1 validation of CLEFT-Q scales for use in children and young adults with facial conditions. J Plast Reconstr Aesthet Surg.

[14] Klassen AF, Riff KWW, Longmire NM, Albert A, Allen GC, Aydin MA, Baker SB, Cano SJ, Chan AJ, Courtemanche DJ, Dreise MM, Goldstein JA, Goodacre TE, Harman KE, Munill M, Mahony AO, Aguilera MP, Peterson P, Pusic AL, Slator R, Stiernman M, Tsangaris E, Tholpady SS, Vargas F, Forrest CR (2018). Psychometric findings and normative values for the CLEFT-Q based on 2434 children and young adult patients with cleft lip and/or palate from 12 countries. Can Med Assoc J.

[15] Hancock SL, Ryan OF, Marion V, Kramer S, Kelly P, Breen S, Cadilhac DA (2020). Feedback of patient-reported outcomes to healthcare professionals for comparing health service performance: a scoping review. BMJ Open.

[16] Apon I, Rogers-Vizena CR, Koudstaal MJ, Allori AC, Peterson P, Versnel SL, Ramirez JP (2022). Barriers and facilitators to the international implementation of standardized outcome measures in clinical cleft practice. Cleft Palate Craniofac J.

[17] Stock NM, Hammond V, Hearst D, Owen T, Edwards Z, Ridley M, Rumsey N (2020). Achieving consensus in the measurement of psychological adjustment to cleft lip and/or palate at age 8+ years. Cleft Palate Craniofac J.

[18] Weidler EM, Britto MT, Sitzman TJ (2021). Facilitators and barriers to implementing standardized outcome measurement for children with cleft lip and palate. Cleft Palate Craniofac J.

[19] Harrison CJ, Rodrigues JN, Furniss D, Swan MC (2022). Response to barriers and facilitators to the international implementation of standardized outcome measures in clinical cleft practice. Cleft Palate Craniofac J.

[20] Weldring T, Smith SMS (2013). Patient-reported outcomes (PROs) and patient-reported outcome measures (PROMs). Health Serv Insights.

[21] PROMIS® Reference Populations.

[22] Varni JW, Magnus B, Stucky BD, Liu Y, Quinn H, Thissen D, Gross HE, Huang I, DeWalt DA (2014). Psychometric properties of the PROMIS ® pediatric scales: precision, stability, and comparison of different scoring and administration options. Qual Life Res.

[23] HL7 International - FHIR Infrastructure Work Group PRO Overview. HL7 FHIR Implementation Guide.

[24] Chalmers RP (2012). mirt: A multidimensional item response theory package for the R environment. J Stat Softw.

[25] Azur Melissa J, Stuart Elizabeth A, Frangakis Constantine, Leaf Philip J (2011). Multiple imputation by chained equations: what is it and how does it work?. Int J Methods Psychiatr Res.

[26] Filzmoser P, Ruiz-Gazen A, Thomas-Agnan C (2013). Identification of local multivariate outliers. Stat Pap.

[27] Chalmers RP (2016). Generating adaptive and non-adaptive test interfaces for multidimensional item response theory applications. J Stat Softw.

[28] Harrison CJ A package for recreating the CLEFT-Q CAT validation study. cleftqCATsim.

[29] Harrison CJ, Rodrigues JN, Furniss D, Swan MC, Klassen AF, Wong Riff KW, Sidey-Gibbons CJ (2021). Optimising the computerised adaptive test to reliably reduce the burden of administering the CLEFT-Q: a Monte Carlo simulation study. J Plast Reconstr Aesthet Surg.

[30] Harrison CJ cleftqCATsim.

[31] Harrison C, Loe BS, Lis P, Sidey-Gibbons C (2020). Maximizing the potential of patient-reported assessments by using the open-source concerto platform with computerized adaptive testing and machine learning. J Med Internet Res.

[32] Tong A, Sainsbury P, Craig J (2007). Consolidated criteria for reporting qualitative research (COREQ): a 32-item checklist for interviews and focus groups. Int J Qual Health Care.

[33] Greenhalgh J, Dalkin S, Gooding K, Gibbons E, Wright J, Meads D, Black N (2017). Functionality and feedback: a realist synthesis of the collation interpretation and utilisation of patient-reported outcome measures data to improve patient care. Health Soc Care Deliv Res.

[34] Harrison C CLEFT-Q-CAT-Score-Checker. GitHub.

[35] Dowrick C, Leydon GM, McBride A, Howe A, Burgess H, Clarke P, Maisey S, Kendrick T (2009). Patients' and doctors' views on depression severity questionnaires incentivised in UK quality and outcomes framework: qualitative study. BMJ.

