# Supplementary material for: The Development, Deployment, and Evaluation of the CLEFT-Q Computerized Adaptive Test: A Multimethods Approach Contributing to Personalized, Person-Centered Health Assessments in Plastic Surgery
Source: J Med Internet Res. 2023 Apr 27;25:e41870. doi: 10.2196/41870 (PMC10185334; doi:10.2196/41870)
Supplement: Multimedia Appendix 1 [file jmir_v25i1e41870_app1.pdf]

# Supplementary Appendix

**Contents**

1.0 Supplementary Methods…………………………………………………………..……………………2

1.1 Supplementary Methods: Rasch Parameterization…………………..……………………………………2

1.2 Supplementary Methods: Missing Data and Outliers in the Validation Dataset……………………………2

1.3 Supplementary Methods: Computerized Adaptive Test Simulation Settings…………………………………2

2.0 Supplementary Results…………………………………………………………………………...5

2.1 Supplementary Results: Missing Data in the Calibration Dataset…………………………………….....5

2.2 Supplementary Results: Rasch Parameterization……………………………………………………...5

2.3 Supplementary Results: Missing Data and Outliers in the Validation Dataset………………………………..5

2.4 Supplementary Results: Computerized Adaptive Test Simulation Settings…………………………………..6

2.5 Supplementary Results: Multistakeholder Workshop….………………………………………..…....6

2.6 Supplementary Results: Concerto Front-End………..…………………………………………………...6

2.7 Supplementary Results: Interview Participants….…………………………………………………………7

2.8 Supplementary Results: Thematic Analysis…….…………………………...………………………...8

References…….………………………………...…………………………………………...………...12

## 1.0 Supplementary Methods

### 1.1 Supplementary Methods: Rasch Parameterization

Rasch parameterization was conducted using *R* version 4.0.0 running under *macOS Mojave 10.14.6* with the mirt package (version 1.32.1) [1].

Rasch models were developed from calibration dataset responses following listwise exclusion of participants with incomplete response sets. To generate Rasch models, we used a fixed-quadrature expectation maximization (EM) algorithm [1]. Before generating Rasch models, the two middle response options in the Speech Distress and Speech Function scales were collapsed, and scoring reversed to represent the current version of the CLEFT-Q [2].

### 1.2 Supplementary Methods: Missing Data and Outliers in the Validation Dataset

Subjects who declined to answer any CLEFT-Q items were not included in the validation dataset. For the included participants, there were missing responses to items in the Speech Distress scale and Social Function scale. In the Speech Distress scale, missing responses existed for one participant who did not answer nine of the ten items. In the Social scale, there were 141 missing responses to item nine, which was added to the scale during data collection. There were also nine missing responses to item seven in the Social Function scale, and all of these occurred at the same study center.

Missing responses were handled through listwise exclusion of respondents with incomplete response sets. For the Social Function scale, we repeated the analysis including all participants, and imputed the 150 missing responses using one iteration of multiple imputation by chained equations and a proportional odds model [3]. Each analysis was performed with and without outliers, who were identified by Mahalanobis distance [4]. Hereafter we describe these repeat analyses as sensitivity-type analyses.

### 1.3 Supplementary Methods: Computerized Adaptive Test Simulation Settings

To perform these computerized adaptive test (CAT) simulations, we developed an R package called cleftqCATsim, which contains 15 functions that allow readers to recreate these experiments with their own data. The key CAT simulation functions serve as convenience wrappers for Phil Chalmers' mirtCAT package [5]. We have made cleftqCATsim available through GitHub with an illustrative vignette [6].

In the CAT simulations, factor scores were calculated for each validation dataset respondent with an expected *a posteriori* approach. Items were selected based on minimum expected posterior variance.

**Supplementary Table 1** Consolidated Criteria for Reporting Qualitative Research checklist[7] for the qualitative component of this study.

| No | Item | Description |
|---|---|---|
| **Domain 1: Research team and reflexivity** | | |
| **Personal Characteristics** | | |
| 1. | Interviewer/facilitator | Interviews were conducted by the manuscript's first author. |
| 2. | Credentials | The interviewer holds a medical degree and is a current doctoral candidate. |
| 3. | Occupation | Honorary plastic surgery registrar and doctoral candidate. |
| 4. | Gender | Male |
| 5. | Experience and training | The interviewer has undertaken a training course on qualitative research with NVivo and has had external supervision from experienced (> 15 years) qualitative researchers. |
| **Relationship with participants** | | |
| 6. | Relationship established | The interviewer met patient-participants during their routine clinic appointments, and has prior relationships with clinician-participants through clinical work. |
| 7. | Participant knowledge of the interviewer | Participants were provided with a Participant Information Sheet which contained detailed information about the study. Participants knew that this work was conducted as part of the interviewer's doctoral thesis. |
| 8. | Interviewer characteristics | The interviewer has led the development of the CLEFT-Q computerized adaptive test as part of his doctoral research. |
| **Domain 2: study design** | | |
| **Theoretical framework** | | |
| 9. | Methodological orientation and Theory | Grounded theory. |
| **Participant selection** | | |
| 10. | Sampling | Patient-participants were purposively selected for diversity in age, gender, ethnicity and diagnosis. Clinician-participants were purposively selected for diversity in occupation. |
| 11. | Method of approach | Participants were approached following routine clinic appointments. All participants had recent (< 7 day) experience of using the CLEFT-Q computerized adaptive test. |
| 12. | Sample size | 6 patient-participants and 4 clinician-participants. |
| 13. | Non-participation | Two patient-participants were unable to attend interviews within 7 days due to logistic and time constraints. |
| **Setting** | | |
| 14. | Setting of data collection | Data were collected either over videoconferencing software in the participant's home, or in the clinical environment following an appointment. |
| 15. | Presence of non-participants | For participants aged < 18 years an adult with parental responsibility was present during the interview. |
| 16. | Description of sample | Sample demographics are presented in Supplementary Table 4. |
| **Data collection** | | |
| 17. | Interview guide | Interview schedules were piloted with one of the interviewer's doctoral supervisors. These are presented in Supplementary Table 2 and Supplementary Table 3. |
| 18. | Repeat interviews | No repeat interviews were conducted. |
| 19. | Audio/visual recording | The interviewer made audio recordings which were transcribed verbatim. |
| 20. | Field notes | Field notes were made where they were required to understand interview responses (e.g. non-verbal responses). |
| 21. | Duration | Interviews ranged in duration from 6 minutes 49 seconds to 20 minutes 25 seconds. |
| 22. | Data saturation | Participants were not deliberately recruited to reach thematic saturation, although no new themes emerged by the final interview. |
| 23. | Transcripts returned | Transcripts were not returned to participants. |
| **Domain 3: analysis and findings** | | |
| **Data analysis** | | |
| 24. | Number of data coders | Data were single coded by the interviewer. |
| 25. | Description of the coding tree | An illustration of the coding tree is provided in Supplementary Figure 3. |
| 26. | Derivation of themes | The following topics were specified *a priori*: experience of the computerized adaptive test's content, experience of the software, barriers to implementing the CLEFT-Q computerized adaptive test, and facilitators to implementing the CLEFT-Q computerized adaptive test. Themes within and in addition to these topics were emergent. |
| 27. | Software | Data were managed in NVivo 1.4. |
| 28. | Participant checking | Participants did not provide feedback on findings. |
| **Reporting** | | |
| 29. | Quotations presented | Quotations are presented in 2.8 Supplementary Results: Thematic Analysis. |
| 30. | Data and findings consistent | Illustrative data are presented 2.8 Supplementary Results: Thematic Analysis. |
| 31. | Clarity of major themes | Major themes are discussed in the main manuscript and are presented in 2.8 Supplementary Results: Thematic Analysis. |
| 32. | Clarity of minor themes | Minor themes are discussed in the main manuscript and are presented in 2.8 Supplementary Results: Thematic Analysis. |

**Supplementary Table 2** Interview schedule for patient-participants.

| Focus area | Opening question and examples of additional probes |
|---|---|
| **Introduction** | Tell me about your last visit to see the cleft team. |
| **Is the CLEFT-Q CAT a worthwhile adjunct to clinical practice?** | What did you think of the CLEFT-Q CAT questionnaire?<br><br>Do you think it changed anything about your conversation with the cleft team?<br><br>What did it change?<br><br>Did it change anything else?<br><br>Did you like completing it?<br><br>Do you think it's a good idea to ask other people to complete the CLEFT-Q CAT at their appointments, just like you did?<br><br>Why do you think that? |
| **How burdensome is the CLEFT-Q CAT?** | How difficult was it to complete the CLEFT-Q CAT?<br><br>What was difficult about it?<br><br>Did it take a long time?<br><br>Was it boring?<br><br>What was boring about it?<br><br>Did it make you tired?<br><br>If you had the choice, would you rather do the CLEFT-Q CAT on an iPad (just like you did) or would you rather have a pen-and-paper version of the questionnaire, with slightly more questions?<br><br>Why? |
| **Facilitators and barriers to CLEFT-Q CAT implementation** | Can you think of anything that might make you less likely to use the CLEFT-Q CAT?<br><br>Can you think of anything that might make you more likely to use the CLEFT-Q CAT? |
| **Areas for CLEFT-Q CAT improvement** | If you could change anything about the CLEFT-Q CAT, what would you change?<br><br>Why?<br><br>Is there anything you really liked about the CLEFT-Q CAT? |

**Supplementary Table 3** Interview schedule for clinician-participants.

| Focus area | Opening question and examples of additional probes |
|---|---|
| **Introduction** | Tell me about your role in the cleft team.<br><br>Have you used the CLEFT-Q CAT a lot? |
| **Is the CLEFT-Q CAT a worthwhile adjunct to clinical practice?** | What do you think of the CLEFT-Q CAT questionnaire?<br><br>Do you think it has changed any aspect of your clinical care, or that of your colleagues?<br><br>What has it changed?<br><br>Has it changed anything else?<br><br>Do you think patients like completing it?<br><br>How useful is it as an adjunct to clinical care?<br><br>Do you think other cleft teams should be using it?<br><br>Why do you think that? |
| **How burdensome is the CLEFT-Q CAT?** | How burdensome is the CLEFT-Q CAT, from your perspective?<br><br>Has it changed your workload, or that of your colleagues?<br><br>In what way?<br><br>Does it make clinics faster or slower?<br><br>Have patients given you feedback about the burden of completing it? |
| **Facilitators and barriers to CLEFT-Q implementation** | Can you think of any barriers to cleft teams implementing the CLEFT-Q CAT?<br><br>Can you think of anything that made it easier or more difficult to implement?<br><br>What advice would you give other cleft teams that are thinking about using the CLEFT-Q CAT? |
| **Areas for CLEFT-Q CAT improvement** | If you could change anything about the CLEFT-Q CAT, what would you change?<br><br>Why?<br><br>Is there anything you really like about the CLEFT-Q CAT? |

## 2.0 Supplementary Results

### 2.1 Supplementary Results: Missing Data in the Calibration Dataset

An analysis of missing items was performed for the calibration dataset and is presented in Sheet 1 of Multimedia Appendix 2. Missing item responses were largely *missing at random* (explainable by other variables). For example, 84% (837/991) of participants missing one or more Jaw scale item(s) were under the age of 12 years. This is because only CLEFT-Q field test participants aged 12-29 years were asked to complete Jaw scale items [2]. Similarly, 50% (307/614) of participants missing one or more School scale items were not attending school (and therefore not administered these items in the CLEFT-Q field test). In the calibration sample, 43% (263/615) of participants missing Speech Distress items, and 39% (263/670) of those missing Speech Function items were born with a cleft lip only, and therefore unlikely to use these subscales in a real-world setting.

### 2.2 Supplementary Results: Rasch Parameterization

Rasch model parameters and fit statistics are presented in Sheet 2 of Multimedia Appendix 2.

### 2.3 Supplementary Results: Missing Data and Outliers in the Validation Dataset

The proportions of outliers for each scale are presented in Sheet 3 of Multimedia Appendix 2.

### 2.4 Supplementary Results: Computerized Adaptive Test Simulation Settings

Full results from the computerized adaptive test (CAT) simulations are presented in Sheet 4 of Multimedia Appendix 2. In this sheet, root mean squared error (RMSE) and 95% limits of agreement are presented as person-location logits, and median values for standard error of measurement are presented for each assessment, with their inter-quartile ranges. This includes all sensitivity-type analyses. In sheet 5 of Multimedia Appendix 2, these results are presented as transformed (0-100) CLEFT-Q scores.

### 2.5 Supplementary Results: Multistakeholder Workshop

Voting results for stopping rules (CAT assessment lengths) at the multistakeholder workshop are presented in Sheet 6 of Multimedia Appendix 2.

### 2.6 Supplementary Results: Concerto Front-End

The patient-facing front-end of the Concerto-based CLEFT-Q CAT app is illustrated in Supplementary Figure 1 and Supplementary Figure 2. Supplementary Figure 1 shows the CLEFT-Q CAT launcher, where relevant scales for the patient can be selected, and Supplementary Figure 2 shows an example item.

**Supplementary Figure 1**

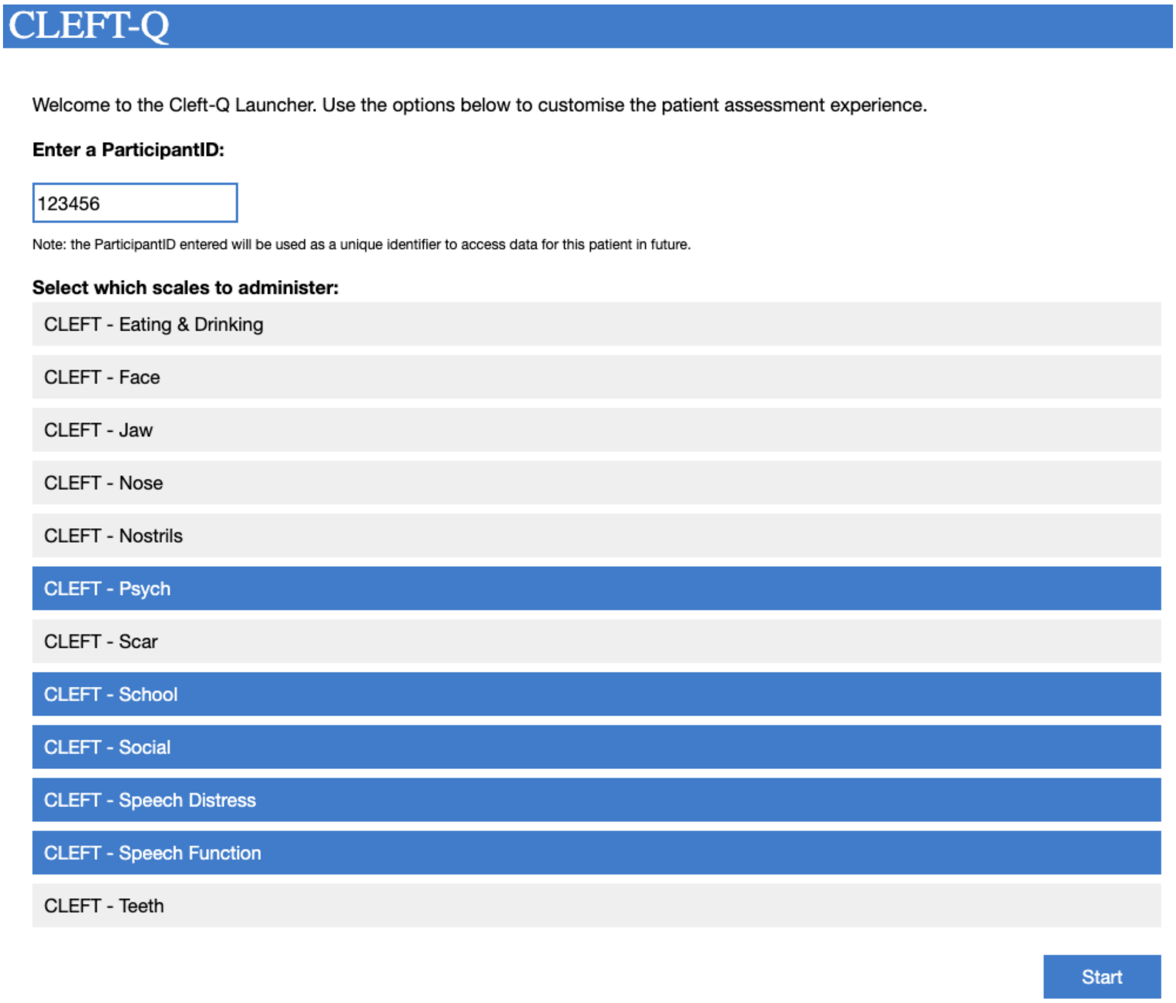


The CLEFT-Q computerized adaptive test launcher.

**Supplementary Figure 2**

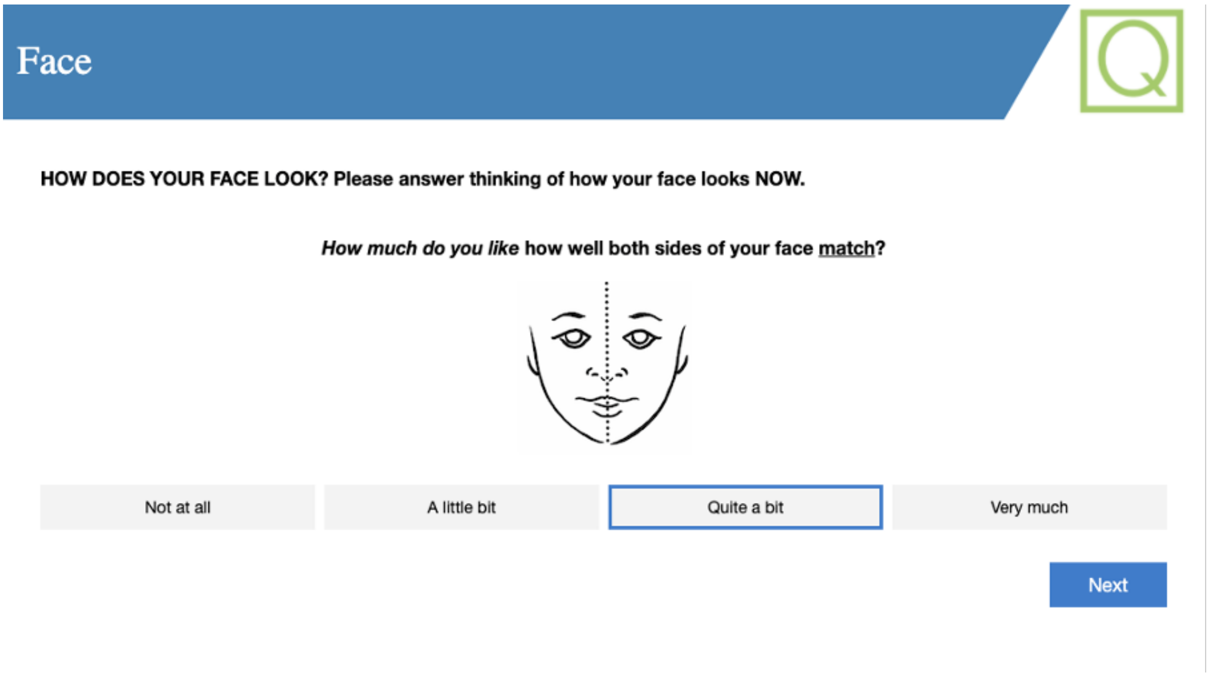


An example item from the CLEFT-Q computerized adaptive test.

## 2.7 Supplementary Results: Interview Participants

Characteristics of interview participants are displayed in Supplementary Table 4.

**Supplementary Table 4.** Interview participant characteristics. UCLP: unilateral cleft lip and palate; UCL: unilateral, isolated, cleft lip; BCLP: bilateral cleft lip and palate.

| **Patients** | | | | |
|---|---|---|---|---|
| **Interview number** | **Gender** | **Cleft type** | **Age** | **Ethnicity** |
| 1 | M | UCLP | 28 | White British |
| 2 | F | UCL | 13 | Kurdish |
| 3 | M | UCLP | 16 | Asian (other) |
| 4 | M | BCLP | 8 | Indian |
| 5 | F | BCLP | 24 | White British |
| 6 | F | BCLP | 18 | British Pakistani |
| **Clinicians** | | | | |
| **Interview number** | **Gender** | **Occupation** | | |
| 7 | M | Surgeon | | |
| 8 | F | Specialist nurse | | |
| 9 | F | Speech and language therapist | | |
| 10 | M | Dentist | | |

## 2.8 Supplementary Results: Thematic Analysis

The interview transcript coding tree is displayed in in Supplementary Figure 3. Below, we present quotes to illustrate themes not included in the main manuscript.

**Supplementary Figure 3**

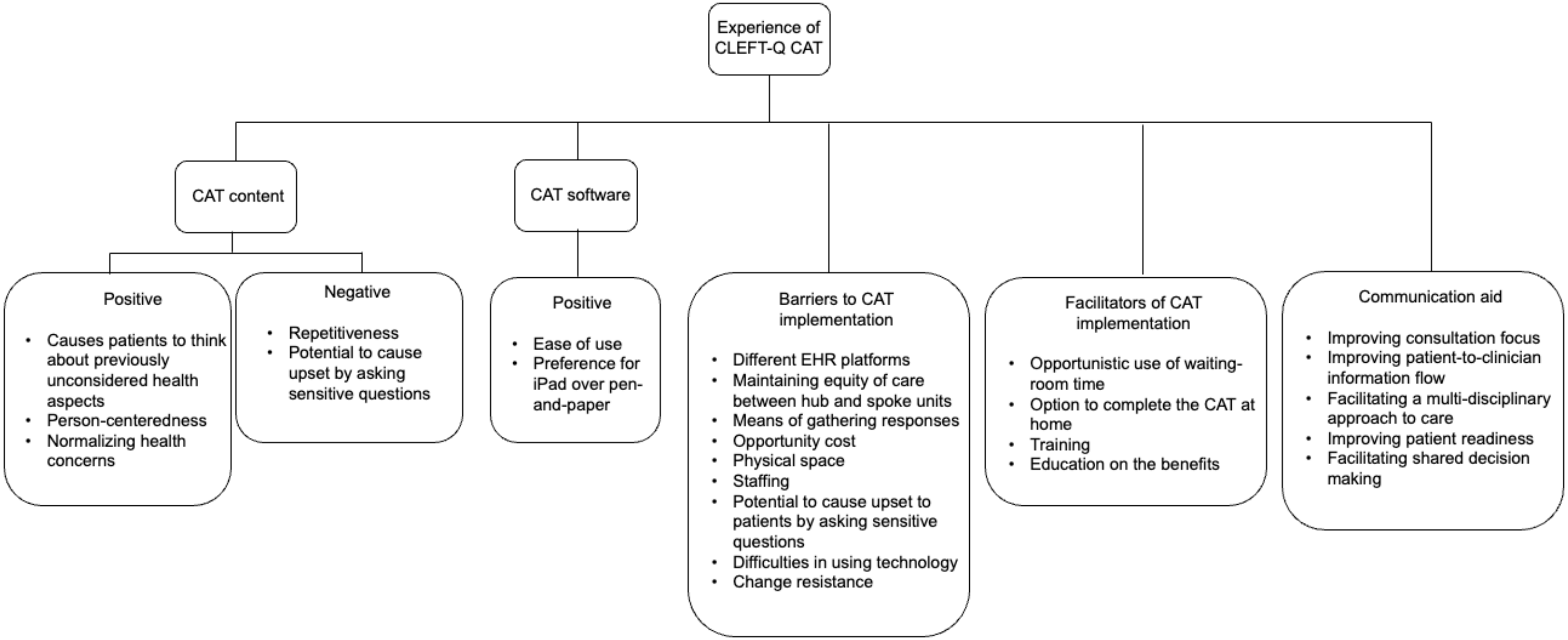


The coding tree from interview transcripts.

**Causing patients to think about previously unconsidered health aspects:**

*"I think it's really detailed, it makes you consider aspects that you don't really consider thinking about too often, so that can help the doctors as well as you" – patient.*

**Person-centeredness:**

*"The questions are I think on topic and are what are relevant to me personally" – patient.*

*"I found that that questionnaire, compared to the paper one I've done in the past, they were similar, but it was more about how I felt, rather than other people" – patient.*

**Normalizing health concerns:**

*"It made it feel way better, like I'm more like other people" – patient.*

**Repetitiveness:**

*"Some of them could have been a bit repetitive. It was almost like the same question but not" – patient.*

**Potential to cause upset to patients by asking sensitive questions:**

*"It's good, because I guess you get to express yourself and say what you feel, I think. But also, I feel like it makes you more conscious about how you look... I didn't find it upsetting personally, but I feel that for other people it probably might be" – patient.*

**Ease of use:**

*"It was really straightforward" – patient.*

*"For me it was pretty quick, so anyone could fill in this form" – patient.*

*"I think the visual appearance [of scores presented as a radar chart] is helpful. Rather than just having scores... you can absorb more information quite quickly" – clinician*

**A preference for using electronic tablets over pen-and-paper:**

*"The iPad is much more convenient [than pen and paper]... you get the results much quicker" – patient.*

**Integration across different electronic health record platforms:**

*"They [other clinicians] will all tend to have different electronic record needs, so it's trying to find something generic, or a platform that can be used across systems, which may be a challenge."* – clinician.

**Maintaining equality between hub and spoke services:**

*"You need to have that equality of care, because the reason for a lot of outlying clinics in cleft is for more deprived patient groups. So, in a way it's even more important that you can roll it out to those groups than it is for the hub patients"* – clinician.

**Means of gathering responses:**

*"Aside from the availability of iPads and then you know the logistics of them missing and signing them in and signing them out, I don't really see any barriers going forwards"* – clinician.

**Opportunity cost:**

*"If you're an adult, you're probably thinking about how you've taken a couple of hours out of your day and you've got your phone on you so you can do some work"* – patient.

Interviewer: *"Anything that would make you want to do it less?"*
Patient: *"Yes"*
Interviewer: *"Like what?"*
Patient: *"Playing games"*
Interviewer: *"So if you could play games instead, you would rather do that? Is that what you're saying?"*
Patient: *Nods and grins cheekily.*

**Physical space:**

*"You need a space for them [the patients] to use [to implement the CLEFT-Q CAT]"* – clinician.

**Staffing:**

*"You need a person who can explain to the patient or the family, what to do [to implement the CLEFT-Q CAT]"* – clinician

**Difficulties in using technology:**

*"I don't know [what might make the CLEFT-Q CAT difficult to implement], it is straightforward to be fair. But it might put them [other patients] off when they see the iPad and they think "oh no", or unless you actually do it, because it was really straightforward, but it could put them off if they see the iPad. Maybe that's older people because a lot of young people know what an iPad is. I wouldn't be put off by doing it" – patient.*

**Change resistance:**

*"When we had similar projects, the barriers tend to be partly personality driven, so some centers will not adopt anything new, or certainly won't adopt anything they didn't develop, almost out of principle. So, there will be some late adopters, where hopefully they'll come on board later"* – clinician.

**Opportunistic use of waiting room time:**

*"If you're a child you're bored and you've got the option of a tablet in front of you, you'd probably do it, personally. It's either that or watching some terrible TV you get in a waiting room… if it's like the 3-4 years I had of brace treatment, I was stuck in the waiting room for 40 minutes, so 10 minutes doing that is probably a welcome distraction" – patient.*

**Option to complete the CAT at home:**

This was seen as a facilitator by some, but not by others:

*"So, personally, I think we had that [the CLEFT-Q CAT] sent out with their appointment, I don't know, a few weeks ahead, that would be really useful because, then, to have that information going in, and we could have had it, if there was anything really significant that was coming up when we reviewed them, then we would be able to discuss that prior to their appointment, so I think, yeah, kind of getting ahead with time would be really good" – clinician*

*"I'm of the opinion that it should be done in the clinical setting with the clinician present at the time, or just before the appointment. Ideally patients would aim to arrive at their appointment a few minutes early anyway, and I think it's a good time to reflect" – clinician.*

**Training:**

*"It would probably be good to have somebody who understands it [the CLEFT-Q CAT] well to come in and have a short training session [with new users]"* – clinician.

**Education surrounding benefits:**

"*A lot of the time you're asked to fill out a questionnaire, and they're [the questionnaire administrators] like 'win a £10 Amazon voucher' – you're not bothered about that. But actually, if they say this will actually really benefit you from a health perspective, and our consultants [attendings] will understand you as a person more, I*

*think they'll go 'actually, yeah, let's not make this a dreaded experience, let's make this an experience we can benefit from'"* – patient.

**References**


1. Chalmers RP. mirt: A Multidimensional Item Response Theory Package for the R Environment. J Stat Softw. 2012;48(6). doi:10.18637/jss.v048.i06

2. Klassen AF, Riff KWW, Longmire NM, et al. Psychometric findings and normative values for the CLEFT-Q based on 2434 children and young adult patients with cleft lip and/or palate from 12 countries. Can Med Assoc J. 2018;190(15):E455-E462. doi:10.1503/cmaj.170289

3. Azur MJ, Stuart EA, Frangakis C, Leaf PJ. Multiple imputation by chained equations: what is it and how does it work?: Multiple imputation by chained equations. Int J Methods Psychiatr Res. 2011;20(1):40-49. doi:10.1002/mpr.329

4. Filzmoser P, Ruiz-Gazen A, Thomas-Agnan C. Identification of local multivariate outliers. Stat Pap. 2014;55(1):29-47. doi:10.1007/s00362-013-0524-z

5. Chalmers RP. Generating Adaptive and Non-Adaptive Test Interfaces for Multidimensional Item Response Theory Applications. J Stat Softw. 2016;71(5). doi:10.18637/jss.v071.i05

6. Harrison CJ. cleftqCATsim. Published 2021. Accessed July 13, 2021. https://github.com/MrConradHarrison/cleftqCATsim

7. Tong A, Sainsbury P, Craig J. Consolidated criteria for reporting qualitative research (COREQ): a 32-item checklist for interviews and focus groups. Int J Qual Health Care. 2007;19(6):349-357. doi:10.1093/intqhc/mzm042
